# Safety and efficacy of quinacrine in human prion disease (PRION-1 study): a patient-preference trial

**DOI:** 10.1016/S1474-4422(09)70049-3

**Published:** 2009-04

**Authors:** John Collinge, Michele Gorham, Fleur Hudson, Angus Kennedy, Geraldine Keogh, Suvankar Pal, Martin Rossor, Peter Rudge, Durre Siddique, Moira Spyer, Dafydd Thomas, Sarah Walker, Tom Webb, Steve Wroe, Janet Darbyshire

**Affiliations:** a National Prion Clinic, National Hospital for Neurology and Neurosurgery, University College London Hospital National Health Service Foundation Trust, London, UK; b Medical Research Council Prion Unit, University College London Institute of Neurology, London, UK; c Department of Neurodegenerative Disease, University College London Institute of Neurology, London, UK; d Medical Research Council Clinical Trials Unit, London, UK

## Abstract

**Background:**

The propagation of prions, the causative agents of Creutzfeldt-Jakob disease and other human prion diseases, requires post-translational conversion of normal cellular prion protein to disease-associated forms. The antimalarial drug quinacrine (mepacrine) prevents this conversion in vitro, and was given to patients with various prion diseases to assess its safety and efficacy in changing the course of these invariably fatal and untreatable diseases.

**Methods:**

Patients with prion disease were recruited via the UK national referral system and were offered a choice between quinacrine (300 mg daily), no quinacrine, or randomisation to immediate quinacrine or deferred quinacrine in an open-label, patient-preference trial. The primary endpoints were death and serious adverse events possibly or probably related to the study drug. This study is registered, ISRCTN 06722585.

**Findings:**

107 patients with prion disease (45 sporadic, two iatrogenic, 18 variant, and 42 inherited) were enrolled, 23 in a pilot study and 84 in the main study. Only two patients chose randomisation; 40 took quinacrine during follow-up (37 who chose it at enrolment). Choice of treatment was associated with disease severity, with those least and most severely affected more likely to choose not to receive quinacrine. 78 (73%) patients died: one randomly assigned to deferred treatment, 26 of 38 who chose immediate quinacrine, and 51 of 68 who chose no quinacrine. Although adjusted mortality was lower in those who chose to take quinacrine than in those who did not, this was due to confounding with disease severity, and there was no difference in mortality between groups after adjustment. Four of 40 patients who took quinacrine had a transient response on neurological rating scales. Only two of 14 reported serious adverse events were judged quinacrine-related.

**Interpretation:**

Quinacrine at a dose of 300 mg per day was reasonably tolerated but did not significantly affect the clinical course of prion diseases in this observational study.

**Funding:**

Department of Health (England); UK Medical Research Council.

## Introduction

No therapeutic intervention prevents or reverses the progressive and ultimately fatal course of human prion diseases, a group of sporadic, acquired, and inherited neurodegenerative disorders characterised clinically by cognitive, neuropsychiatric, and motor dysfunction.^1^ Prion diseases are highly heterogeneous in clinical and pathological phenotypes. The commonest form is sporadic Creutzfeldt-Jakob disease (CJD), which affects about 1–2 people per million annually worldwide and presents most commonly with rapidly progressive dementia and a median survival of 4–6 months.^1^ Acquired prion diseases have developed after treatment with human cadaveric pituitary derived hormones, surgery involving contaminated neurosurgical instruments, dura-mater grafts or corneal transplants, mortuary feasts in Papua New Guinea, and, in variant CJD, through exposure to prions from cows with bovine spongiform encephalopathy and through blood transfusion.^2^, ^3^ Inherited prion diseases occur as a result of one of the more than 30 known mutations in the prion protein gene, *PRNP*. These dominantly inherited disorders of high penetrance account for about 15% of identified human prion disease, presenting with various clinicopathological syndromes including classic CJD, Gerstmann-Sträussler-Scheinker disease, and fatal familial insomnia.^1^

The neuropathological processes underlying these diseases are associated with post-translational conversion of normal cellular prion protein, PrP^C^, to abnormal disease-associated forms of the same protein, PrP^Sc^, through conformational change and aggregation. Many experimental therapeutic approaches have been assessed with prevention of PrP^Sc^ formation in cell and animal models as a surrogate marker, even though accumulation of PrP^Sc^ does not necessarily correlate with neurotoxicity.^4^, ^5^ In prion-infected cultured mouse cells, several compounds, including quinacrine (mepacrine), block PrP^Sc^ production.^6^, ^7^ Extensive clinical experience of treating both malaria and rheumatoid arthritis has shown that oral quinacrine is safe and can cross the blood–brain barrier; therefore, the drug is potentially useful in the treatment of human prion disease.^7^ Interest in this potential treatment grew, and some patients requested immediate access to quinacrine. The Chief Medical Officer asked the Medical Research Council to sponsor a clinical trial in prion disease and to investigate any therapeutic potential of quinacrine. While the study was being prepared, a pilot study was developed allowing all patients requesting quinacrine to receive it with appropriate medical monitoring.

Given the relentless and often rapid progression, invariably fatal outcome, and lack of other treatment options for prion neurodegeneration, randomisation to placebo was not likely to be acceptable to many patients or their families and carers. Because many patients with human prion disease are incapacitated at the time of diagnosis, family and carers have an important role in decisions about participation in a research study. A formal consultation process in the UK with patients, families, carers, and representatives of patients was done to develop an acceptable study protocol.^8^ After this consultation, PRION-1 was designed as a patient-preference trial in which patients were given the option of random treatment allocation^9^ to investigate the use of quinacrine in all forms of prion disease in the UK.

## Methods

### Patients

To ensure that PRION-1 enrolled a sufficient number of patients, recruitment of a high proportion of all UK patients with prion disease was needed because of the rarity of the diseases. A national referral system was set up to recruit patients while continuing to support ongoing epidemiological studies and surveillance. In 2004, all UK neurologists were asked by the Chief Medical Officer to refer all patients with suspected prion disease jointly to the National CJD Surveillance Unit (Edinburgh, UK) and to the National Prion Clinic (London, UK), enabling participation in research, including the PRION-1 trial. Before the formal launch of PRION-1, patients attending the National Prion Clinic could enter a pilot phase of the trial in which randomisation was not offered. Patients with any form of human prion disease who met standard diagnostic criteria^10^ and who were aged 12 years or older were eligible. Individuals with known hypersensitivity to quinacrine, who had taken any other potential antiprion drug within the past 2 months, or who clinicians judged to be in a terminal disease state were ineligible. All referred patients or their carers were contacted to ask if they would agree to a home or clinic screening visit from the PRION-1 team. Patients were seen at enrolment and subsequently either at the National Prion Clinic or at their homes by the same members of the PRION-1 clinical team. The PRION-1 trial was approved by the Eastern Multicentre Research Ethics Committee. All patients gave consent, or assent was provided by a family member or independent neurologists.

### Study design

**Figure 1:**
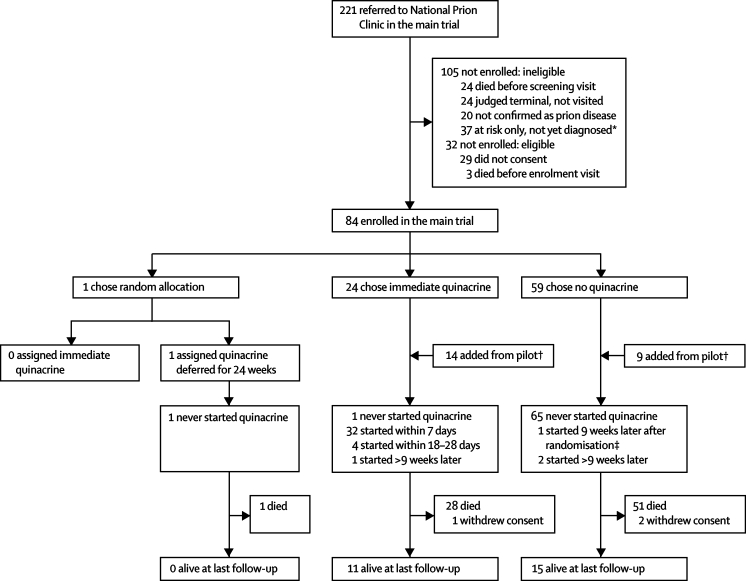
Trial profile *Relative with symptomatic inherited prion disease or recipient of blood transfusion. †From September, 2001–June, 2002, six patients received open-label quinacrine in an initial pilot study, and from August, 2002, to March, 2004, 17 more were offered quinacrine or no quinacrine in an extended pilot study (total 23 patients). ‡One originally chose not to take quinacrine but later agreed to randomisation 9 weeks after enrolment and was allocated immediate quinacrine.

PRION-1 was an open-label patient-preference trial in which patients were given the option of random allocation to treatment, with a planned recruitment of 160 individuals over 2 years and a minimum follow-up of 1 year (figure 1). The objective was to obtain data on the effect of quinacrine in human prion disease, from a randomised comparison where acceptable and otherwise from observational comparisons. Patients who were willing (or, in the case of incapacity, those whose advocates or relatives were willing for them to be) were randomly allocated: either to immediate or deferred (for 6 months) quinacrine. Those patients, or their relatives or advocates in cases of incapacity, who were not willing to be randomised could choose either to take quinacrine immediately or not.

Patients were assessed at baseline and follow-up at 1, 2, 4, and 6 months and then after intervals of 3 months. Neurological assessments and investigations included full blood count and biochemistry, a standardised clinical and neurological examination, cognitive assessment (mini mental state examination [MMSE], range 0–30;^11^ cognitive component of the Alzheimer's disease assessment scale [ADAS-cog], range 0–75;^12^ Glasgow coma score [GCS], range 3–15),^13^ clinical dementia rating (CDR sum of scores, range 0–18),^14^ the clinician-interview-based impression of change plus carer input (CIBIC-P-plus) component of the clinician's global impression of change (range 1–7),^15^ brief psychiatric rating scale (BPRS version 4, range 24–168),^16^ Rankin scale (range 1–5),^17^ and Barthel activities of daily living index^18^ (range 0–20).

Randomisation was stratified by inherited versus other prion disease; pre-prepared lists were computer generated and sequentially numbered and securely incorporated within the trial database. To randomly assign treatment, PRION-1 clinicians were to contact the Medical Research Council Clinical Trials Unit by telephone. Quinacrine was given orally with a loading dose of 1 g over 24 h (200 mg every 6 h), followed by 100 mg three times daily. Because quinacrine produces a characteristic skin discolouration, a double-blind comparison was not possible, and all patients, carers, clinicians, and nurses therefore knew whether or not patients were receiving quinacrine. A standardised digital video recording, converted to black and white, of the clinical and neurological examination was taken by the trial physician and scored by an independent assessor who was unaware of the treatment allocation. Video recordings were reviewed in random order for each patient.

The primary efficacy endpoints were death and treatment response, defined in the protocol as either clinical improvement or lack of deterioration on the digital recording of the neurological examination, the CIBIC-P-plus, and the BPRS. Because the BPRS could be recorded at only 28% of assessments, and because it was commonly impossible to assign an overall assessment of response across the many domains of the digital recording, a revised definition of response was agreed by the trial steering committee, namely improvement on two or more clinical rating scales with no deterioration on all the other rating scales used at the same time point where improvement or lack of deterioration had been predefined for each rating scale in the protocol. The primary safety endpoint was any serious adverse event not known to be or likely to be related to prion disease and considered possibly or probably related to quinacrine, with serious events defined as fatal, life threatening, requiring or increasing the length of time in hospital, or resulting in persistent or significant disability or other important medical events. Serious adverse events in the PRION-1 trial (from April, 2004) were independently reviewed by a member of the trial steering committee. Toxicity grades were defined according to the National Cancer Institute common toxicity criteria (version 2.0)^19^ with minor modifications. There was no requirement to report a laboratory grade 4 adverse event as a serious adverse event unless it met the clinical criteria for seriousness.

### Statistical analysis

Kaplan–Meier plots, log-rank tests, and Cox proportional hazards models were used to compare groups for time-to-event outcomes. Categorical variables were compared with exact tests, and continuous variables with *t* tests and rank-sum tests. Baseline values were those recorded before and nearest to enrolment (all within the preceding 9 days). The closest measurement to the scheduled assessment week within equally spaced time windows was used subsequently. Missing MMSE or CDR values were imputed as the worst score (0 or 18, respectively) if GCS was 14 or lower, and missing GCS was imputed as the best score (15) if MMSE was 5 or higher. Logistic models for initial preference of quinacrine versus no quinacrine used multiple imputation with chained estimation combining results from 50 imputations for remaining missing baseline values (in six of 101 patients) with Rubin's rules^20^ or analyses of survival. Follow-up time for each patient was split into before and after quinacrine periods (cohort analysis), counting time from enrolment and using late entry for patients initiating quinacrine after enrolment. For adverse events, time at risk was classified by whether or not patients were on quinacrine or had taken it in the last 30 days. All p values are two-sided. The original sample size for the randomised comparison of 87 patients was designed to provide at least 80% power to detect a reduction in 2 year mortality from 50% to 22% (hazard ratio 0·35, two-sided α=0·05).

This study is registered, ISRCTN 06722585.

### Role of the funding source

The sponsor had no role in the study design, data collection, data analysis, data interpretation, or writing of the report. The corresponding author had full access to all the data in the study and had final responsibility for the decision to submit for publication.

## Results

From September, 2001, to June, 2002, six patients received open-label quinacrine in the pilot study; from August, 2002, to March, 2004, 17 were offered a choice of quinacrine or no quinacrine in an extension of this pilot study. From April, 2004, to August, 2006, 84 patients enrolled in the main PRION-1 trial. All patients were followed up until March 31, 2007. 107 patients, 50 (47%) of whom were male, were recruited in the pilot or main study (figure 1). 38% of referred and 72% of eligible patients were enrolled. Only one patient chose random allocation of treatment at enrolment and was allocated to deferred quinacrine but chose not to start quinacrine at 24 weeks. A second patient chose not to take the drug at enrolment, but subsequently opted for random allocation at week 9; this patient commenced immediate quinacrine. Consequently PRION-1 is primarily an observational study of patients who chose the drug or did not.

Table 1 lists the characteristics at enrolment of all patients according to the patient's initial preference for quinacrine or no quinacrine when this choice was offered. A definitive diagnosis (genetic mutation or tissue examination including tonsil biopsy at screening or post-mortem examination) was made in 78 (73%) patients: none was wrongly diagnosed. In total, 38 patients chose quinacrine at enrolment, of whom 37 received the drug. 69 patients chose no quinacrine initially; three of them took the drug at some point during follow-up (figure 1). One patient who chose quinacrine had previously taken the drug for 3 weeks from 5 weeks before enrolment and was enrolled in error. This patient was included in the analysis.

**Table 1:** Characteristics at enrolment: overall and according to initial choice of quinacrine or not

		All patients*	**Chose quinacrine at enrolment**	**Chose no quinacrine at enrolment**†	**p value**‡	**Chose quinacrine *vs* no quinacrine OR (95% CI; p)**§
Enrolled		107 (100%)	32 (100%)	69 (100%)		
Studies					0·16	
	Pilot study	23 (22%)	8 (25%)	9 (13%)		
	Main study	84 (78%)	24 (75%)	60 (87%)		
Type of prion disease					0·06	..
	Sporadic	45 (42%)	8 (25%)	36 (52%)		
	Iatrogenic	2 (2%)	1 (3%)	1 (1%)		
	Variant	18 (17%)	6 (19%)	10 (14%)		
	Inherited	42 (39%)	17 (53%)	22 (32%)		
Median age (years; range)		56 (14–82)	55 (17–75)	58 (19–82)	0·18	..
	Non-inherited disease	60 (14–82)	60 (17–75)	62 (19–82)		
	Inherited disease	43 (32–72)	53 (32–65)	41 (32–72)		
Median time (months) since first symptoms¶ (range)		10 (1–140)	13 (2–118)	8 (1–140)	0·04	..
	Non-inherited disease	7 (1–50)	9 (2–19)	7 (1–50)		
	Inherited disease	26 (3–140)	25 (4–118)	27 (3–140)		
Barthel index						1·89 (0·97–3·62; 0·06)
	Number assessed	95 (89%)	28 (88%)	67 (97%)	0·08	
	Median (IQR)	4 (11–17)	14 (5–18)	2 (0–12)	0·0007	
MMSE						1·29 (1·06–1·57; 0·01)
	Number assessed	58 (54%)	26 (81%)	27 (39%)	0·0001	
	Median (IQR)	20 (15–25)	23 (18–26)	18 (14–25)	0·20	
	Median observed/imputed value (IQR)‖	7 (0–21)	22 (7–25)	0 (0–15)	<0·0001	
CDR						0·64 (0·44–0·92; 0·02)
	Number assessed	75 (70%)	27 (84%)	42 (61%)	0·02	
	Median (IQR)	8 (4–12)	6 (2–8)	9 (6–13)	0·01	
	Median observed/imputed value (IQR)‖	11 (6–18)	7 (3–10)	16 (8–18)	0·0007	
GCS						..
	Number assessed	74 (69%)	19 (59%)	54 (78%)	0·06	
	Median (IQR)	12 (10–14)	15 (11–15)	12 (9–14)	0·001	
	Median observed/imputed value (IQR)‖	14 (11–15)	15 (14–15)	13 (10–15)	<0·0001	
ADAS-cog						
	Number assessed	37 (35%)	20 (62%)	17 (25%)	0·0004	
	Median (IQR)	17 (8–29)	18 (8–26)	16 (8–32)	0·95	..
BPRS						..
	Number assessed	37 (35%)	19 (59%)	18 (26%)	0·002	
	Median (IQR)	33 (30–40)	37 (30–40)	32 (30–40)	0·46	
Rankin scale						
	Number assessed	105 (98%)	32 (100%)	67 (97%)	1·00	
	No or slight symptoms (1/2)**	13 (12%)	5 (16%)	8 (12%)	0·0007	1·00 (0·09)
	Moderate disability (3)	21 (20%)	11 (34%)	9 (13%)		3·21 (0·71–14·5)
	Moderate to severe disability (4)	31 (30%)	12 (38%)	15 (22%)		5·20 (0·90–30·0)
	Severe disability (5)	40 (38%)	4 (12%)	35 (52%)		1·52 (0·17–13·5)
CIBIC-P						
	Number assessed	105 (98%)	31 (97%)	68 (99%)	0·54	
	Normal or borderline (1/2)**	7 (7%)	1 (3%)	6 (9%)	0·0001	1·00 (0·07)
	Mildly ill (3)	11 (10%)	7 (23%)	4 (6%)		12·8 (1·06–155)
	Moderately ill (4)	23 (22%)	11 (35%)	10 (15%)		11·6 (1·04–130)
	Markedly ill (5)	26 (25%)	9 (29%)	14 (21%)		14·3 (0·91–222)
	Severely ill or the most ill (6/7)	38 (36%)	3 (10%)	34 (50%)		3·39 (0·13–85·5)

Data are number (%) unless otherwise stated. MMSE=mini-mental state examination. CDR=clinical dementia rating. GCS=Glasgow coma scale. ADAS-cog= Alzheimer's Disease Assessment scale cognitive component. BPRS=brief psychiatric rating scale. CIBIC-P=clinician interview-based impression of change plus carer input.

* Includes the first six patients who received quinacrine in a pilot study without the option of no quinacrine who are excluded from comparisons according to choice.

† One patient who chose to be randomised at enrolment is included in the chose no quinacrine group (the other randomised patient chose no quinacrine at enrolment, see Results).

‡ Univariate p values from exact tests (categorical) or ranksum (continuous) assessing the effect of each factor on choice of quinacrine or no quinacrine.

§ Multivariate independent predictors; the best multivariate logistic models adjusted for baseline Rankin score or CIBIC-P and one of Barthel index, MMSE, and CDR; numbers were too small to discriminate further between these predictors; effect on choice of quinacrine versus no quinacrine shown for Rankin and MMSE (OR per 3 units higher) from model including Rankin and MMSE, for CDR and Barthel index (OR per 3 units higher) from model including Rankin and CDR or Barthel index, and for CIBIC-P from a model including CIBIC-P and MMSE (similar results were obtained for other models combining these factors), absence of OR means no evidence of a independent contribution of this factor (p>0·10).

¶ Excluding six patients asymptomatic at enrolment (one chose quinacrine, five chose no quinacrine).

‖ Methods for imputation strategy; number of imputed baseline values: MMSE 48, CDR 30, GCS 32.

** Percentages of non-missing values.

In univariate analyses, MMSE was the most important predictor of choice of quinacrine, with all other neurological rating scales and inherited versus other human prion disease also significant univariable predictors. However, these baseline factors were all strongly associated with each other, and the number of participants was too small to unambiguously identify independent predictors of opting for quinacrine. The best models indicated that both the most and least severely affected patients (by either CIBIC-P or Rankin scale) were least likely to choose quinacrine. Those patients with better MMSE, Barthel index, or CDR scores were independently significantly more likely to choose the drug than were those with similar scores on the CIBIC-P or Rankin scale (table 1). Thus, unadjusted comparisons of patients choosing to take or not take quinacrine are confounded by disease severity.

**Figure 2:**
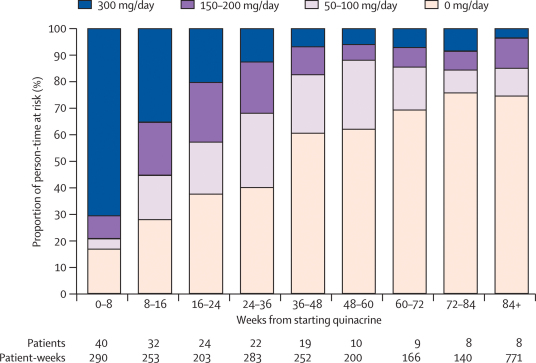
Quinacrine use after initiation

40 patients received quinacrine, with four starting 9 weeks or more after enrolment (figure 1). Overall, 46% of person-time at risk before death or end of study was spent on quinacrine (at any dose) after starting it (figure 2), with a Kaplan–Meier median 7·4 months (1·7–14·8) to permanent discontinuation. Five (12%) patients were still alive and taking quinacrine at the end of the study (three on 100 mg daily, two on 200 mg daily). 24 (60%) patients reduced drug dose (12 alive at end of study including the five still taking quinacrine), 11 (28%) permanently discontinued quinacrine 300 mg daily (three alive at end of study), and five (12%) continued on their initial 300 mg dose until they died. Two patients who chose not to receive the study drug took other putative antiprion drugs during PRION-1: one took flupirtine from 3 weeks to 7 weeks after enrolment, and the other took pentosan polysulfate from 4 weeks to 60 weeks; when they withdrew consent.

78 (73%) patients died (34 within 6 weeks of enrolment), 26 (68%) of 38 who initially chose quinacrine (one died before starting the drug) and 52 (75%) of 69 who initially chose no quinacrine (including the one patient who was randomised to no quinacrine). The median follow-up in the 29 patients not known to have died was 16 months (IQR 12–25); three withdrew consent.

**Figure 3:**
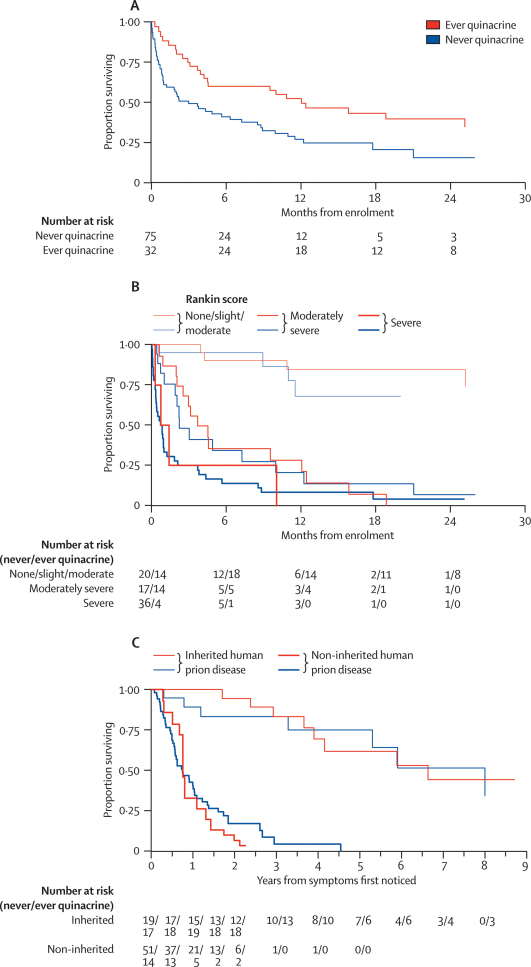
Survival Unadjusted survival from enrolment (A). Survival from enrolment by baseline Rankin score (B). Survival from first symptoms by type of human prion disease (C).

Although unadjusted mortality seemed lower in patients choosing to take quinacrine (table 2, figure 3), this was a consequence of substantial confounding between choosing treatment and baseline characteristics, particularly inherited versus other types of prion disease and scores on neurological rating scales, which were all strong independent predictors of mortality (p<0·0001, table 2). After adjusting for the low mortality risk in women, young patients, those with inherited prion disease, and those with better clinical or neurological status at baseline (either by Rankin scale, CDR, or Barthel index), there was no significant difference in survival (figure 3) between patients who took quinacrine and those who did not after enrolment, stratified by baseline Rankin scake (hazard ratio [HR] 0·87, 95% CI 0·49–1·52; p=0·62) or survival from reported time of first symptoms stratified by type of prion disease (1·14, 0·69–1·89; 0·61).

**Table 2:** Predictors of mortality

		**Univariate effect on risk of mortality**		Multivariate effect on risk of mortality*(model 1)		Multivariate effect on risk of mortality*(model 2)	
		OR (95% CI)	p value	OR (95% CI)	p value	OR (95% CI)	p value
Quinacrine use†		0·52 (0·31–0·86)	0·01	1·10 (0·60–2·01)	0·76	0·98 (0·56–1·71)	0·94
Sex		0·71 (0·45–1·12)	0·14	0·42 (0·25–0·72)	0·002	0·47 (0·28–0·77)	0·003
Age (per 10 years)		1·68 (1·40–2·01)	<0·0001	1·31 (1·10–1·56)	0·002	1·32 (1·12–1·57)	0·001
Months since first symptoms‡ (per 12 months)		0·70 (0·58–0·85)	0·0003	..	..	..	..
Inherited prion disease		0·13 (0·07–0·24)	<0·0001	0·26 (0·13–0·53)	0·0002	0·24 (0·12–0·47)	<0·0001
Period of study: pilot		0·35 (0·18–0·68)	0·02	..	..	..	..
	April, 2004, to March, 2005 (main trial)	1·00					
	April, 2005, to December, 2005	0·82 (0·47–1·44)					
	2006	0·85 (0·44–1·64)					
MMSE (per 3 units higher)		0·79 (0·74–0·85)	<0·0001	..	..	..	..
Rankin scale			<0·0001	0·03		..	..
	No or slight symptoms	1·00		1·00			
	Moderate disability	1·03 (0·26–4·11)		0·45 (0·11–1·89)			
	Moderate to severe disability	7·35 (2·29–23·6)		1·45 (0·35–6·08)			
	Severe disability	14·9 (4·65–47·5)		2·52 (0·56–11·3)			
GIC			<0·0001	..	..	..	..
	Normal, borderline, mildly ill§	1·00					
	Moderately ill	2·33 (0·78–6·96)					
	Markedly ill	8·54 (3·05–23·9)					
	Severely ill	17·1 (5·97–49·2)					
	Amongst the most ill	18·0 (6·18–52·3)					
Barthel index (per 3 units higher)		0·65 (0·57–0·73) <0·0001	..	..	..	0·80 (0·67–0·96)	0·02
CDR (per 3 units higher)		1·68 (1·46–1·93) <0·0001	..	1·25 (1·02–1·52)	0·03	1·19 (0·98–1·45)	0·08
GCS (per 3 units higher)		0·39 (0·31–0·49) <0·0001	..	..	..	..	..

MMSE=mini-mental state examination. GIC=global impression of change. CDR=clinical dementia rating, GCS=Glasgow coma scale.

* Unstratified adjusted model: numbers were too small to unambiguously identify independent predictors.

† Time-updated variable: whether a patient had already taken quinacrine in PRION-1 versus never or not yet initiated.

‡ For asymptomatic patients (n=6), months since first symptoms imputed as 2 years more than the maximum observed.

§ All patients classed “normal” or “borderline” by GIC (n=7) were alive at the end of the study, and therefore to fit models this group is combined with the mildly ill (n=11). Absence of OR means no evidence of a significant independent contribution of this factor (p>0·10).

Defining response as improvement on two or more neurological rating scales without deterioration in any other scales measured at the same time point (improvement or deterioration predefined in the protocol), four of 40 patients who took immediate quinacrine had a transient response at one or two visits after treatment started (all four experienced quinacrine-related toxicity), compared with one of 71 patients before quinacrine initiation (table 3). This patient had improved from 24 weeks after enrolment throughout follow-up to week 113 when last seen alive, suggesting that the data recorded at the enrolment visit might inaccurately represent baseline status for this patient (table 4).

**Table 3:** Overall response based on neurological rating scales

	**After enrolment and before quinacrine initiation**			**After quinacrine initiation**			**After quinacrine initiation: adverse-event reported**		
	Total	Last seen alive	Last seen dead	Total	Last seen alive	Last seen dead	Total	Last seen alive	Last seen dead
Response	1 (0/1)*	1 (0/1)	..	4 (1/3)†	3 (0/3)	1 (1/0)	4 (1/3)	3 (0/3)	1 (1/0)
Stable	17 (7/10)	10 (1/9)	7 (6/1)	4 (2/2)	1 (0/1)	3 (2/1)	3 (2/1)	1 (0/1)	2 (2/0)
Deterioration	24 (15/9)	7 (3/4)	17 (12/5)	28 (10/18)	11 (1/10)	17 (9/8)	24 (7/17)	11 (1/10)	13 (6/7)
Baseline data only*	29 (27/2)	..	29 (27/2)	4 (4/0)	..	4 (4/0)	..	..	..
Total	71 (49/22)	18 (4/14)	53 (45/8)	40 (17/23)	15 (1/14)	25 (16/9)	31 (10/21)	15 (1/14)	16 (9/7)

Data are number (number with non-inherited disease/number with inherited disease). For each individual rating scale, response was predefined in the protocol as an increase of 3 units on the mini-mental state examination, 2 units on the Barthel index, or 2 units on the Glasgow coma scale, and a decrease of 1 unit on the Rankin scale, 3 units on clinical dementia rating (CDR), 1 unit on global impression of change (GIC), 10 units on Alzheimer's Disease Association-cognitive component, and 6 units on the brief psychiatric rating scale. Deterioration was defined as the inverse (decrease or increase respectively); those not meeting criteria for either response or deterioration were defined as stable. Overall response is defined as response on two or more of the neurological rating scales, without deterioration on any other scores measured at the same timepoint at any time during follow-up.

* Response compared with baseline at all visits from week 37 through 113 (last seen alive).

† Response at one or two visits only followed by deterioration before date last seen alive or death.

**Table 4:** Details of responders

	**Quinacrine**	**Human prion disease**	Response at weeks*	**GCS range 3–15**	**Rankin range 1–5**	**GIC range 1–7**	**BPRS range 24–168**	**Barthel range 0–20**
Died week 52	Yes	Sporadic	4	11→13	4→3	..	..	..
Alive week 167	Yes	Inherited	17, 40†	..	3→2	4→3	..	..
Alive week 104	Yes	Inherited	11	..	3→2	..	42→25	..
Alive week 65	Yes	Inherited	4, 28‡	..	3→2	4→3	39→25	..
Alive week 113	No	Inherited	24–113	..	4→3	4→2-3	..	10→12–15

High scores are favourable on Glasgow coma scale and Barthel index and low scores are favourable on Rankin scale, global impression of change (GIC), and brief psychiatric rating scale.

* Weeks after quinacrine initiation for those with response on quinacrine, otherwise weeks from enrolment.

† One intervening visit without response.

‡ Three intervening visits without response. Missing data indicate no change or value not measured.

14 serious adverse events were reported in 11 patients, nine in seven patients taking quinacrine at the time or in the preceding 30 days (table 5). Ten serious adverse events (five on study drug) required hospital admission. At independent review, seven of the 13 serious adverse events during the main trial phase were judged definitely or probably related to prion disease, and only two of the remaining six were thought possibly or probably related to quinacrine (a tonic-clonic seizure on day 3 and aspiration pneumonitis on day 6 of treatment). 17 grade 3 or 4 adverse events were reported in 16 patients on quinacrine, 11 of which were thought possibly or probably related to the drug including five raised results on liver-function tests and two rashes, compared with five grade 3 or 4 events in four patients off quinacrine. However, dose modification or discontinuation was more common after mostly low-grade adverse events (total 53 adverse events) than after serious events, most commonly associated with abnormal results of liver-function tests (20 patients), rashes (nine), and nausea (seven).

**Table 5:** Grade 3 or 4 and serious adverse events and reactions, and adverse events leading to quinacrine discontinuation or dose reduction

	**Reported serious adverse events**		Independently adjudicated*serious adverse events		**Reported grade 3 or 4 adverse events**		**Reported adverse events (any grade) leading to quinacrine discontinuation or dose reduction**
	On†	Off	On†	Off	On†	Off	
Rash					2 (2)		9
Yellow skin							1
Dry skin							1
Grand mal convulsion or seizure	2 (2)		1 (1)		2 (2)		1
Haematemesis	1 (1)						2
Haematuria and haematemesis	1 (1)		1		1 (1)		1
Abdominal wall abscess		1		1		1	
Diarrhoea							1
Nausea					1 (1)		3
Nausea and delirium							1
Nausea and diarrhoea							1
Nausea and diarrhoea and high alanine aminotransferase concentrations							1
Nausea and paraesthesia							1
Vomiting and dysphagia	1		1		1		
Vomiting and aspiration pneumonitis	1 (1)		1 (1)				1
Aspiration pneumonia	2				2		1
Liver-function test abnormal					2 (2)		2
Alanine aminotransferase increased					3 (3)		16
Aspartate aminotransferase increased							2
Alkaline phosphatase increased							2
Lower-respiratory-tract infection	1				1		1
Pneumonia bacterial and lung consolidation		1				1	
Urinary-tract infection bacterial					1		
Suicide attempt		1		1		1	
Fracture and pneumothorax traumatic					1		
Laceration		1				1	
Aggression		1				1	
Abnormal behaviour							1
Agitation and somnolence							1
Other‡							3
Total events	9 (5)	5	4 (2)	2	17 (11)	5	53
Rate per 100 patient-months	2·9 (1·6)	0·7	1·8 (0·9)	0·3	5·5 (3·6)	0·7	25·4
Total patients	7 (4)	4	4 (2)	2	16 (10)	4	30

Data are number of events, with those judged as probably or possibly related to quinacrine in parentheses. Difference between on and off quinacrine in rate of all reported serious adverse events (p=0·006) and all grade 3/4 adverse events (p<0·0001). All reported serious adverse events were also grade 3 or 4 events except for haematemesis (grade 2) and vomiting and aspiration pneumonitis (reported as grade 1 vomiting and dyspnoea but judged grade 4 aspiration pneumonitis at independent review).

* Serious adverse events during the main trial were reviewed by an independent trial physician (one aspiration pneumonia during the pilot study was not reviewed).

† While taking quinacrine or within 30 days of stopping.

‡ Acquired pigmented retinopathy, abnormal EEG, and hyponatraemia.

## Discussion

After adjusting for the substantial differences between patients who chose to take quinacrine or not, we did not find any evidence that oral quinacrine at a dose of 300 mg a day increased the length of survival of patients with prion disease.

The aims of PRION-1 were to assess the risks and benefits of quinacrine in human prion disease and to establish a framework for the clinical assessment of future therapeutic options. As the first major prospective study with longitudinal assessments in human prion disease in the UK, PRION-1 showed that national recruitment and retention is feasible and acceptable to patients and carers. Availability of dedicated clinical teams to visit patients in their local environment and to respond rapidly to any referrals was important because sporadic and acquired prion diseases are severely debilitating and rapidly progressive. However, PRION-1 highlighted the difficulty of randomised controlled trials in human prion disease. Only two of 84 patients or their carers agreed to randomisation, even though the design of the trial meant that all participants would receive quinacrine either immediately or after an interval in the deferred arm. Affected patients, or the families and carers of individuals who lacked capacity to give consent, overwhelmingly preferred to make decisions on treatment rather than agree to randomisation. Patients and their carers faced with the prospect of a rapidly progressive fatal disease were unwilling to accept the possibility of randomisation to deferred treatment, whereas many families caring for a severely demented and physically incapacitated family member were not prepared to accept a therapeutic intervention that, at best, was expected to slow or halt disease progression with little or no prospect of reversing established neurological damage. Because no potential treatment is likely to reverse neuropathology or clinical status in severely affected individuals, future trials aimed at mild to moderately affected patients might offer the best prospect of acceptance of random treatment allocation and therefore the ability to reliably assess a new treatment.^21^ There was equipoise, at least at the population level if not at an individual level, in patients who were mild to moderatly affected in PRION-1, in that about half chose quinacrine and half chose no quinacrine. A further complexity was yellow skin discolouration, which prevented a fully blinded study.

PRION-1 was essentially an observational study of patients choosing to take quinacrine or not, in which we identified strong determinants of this choice, the most important being severity of disease. Although quinacrine was associated with a raised incidence of adverse events, most were mild and led only to dose reduction or discontinuation. These findings are consistent with a case–control study in which the survival of 30 patients with sporadic CJD who received quinacrine was not significantly different from that in control individuals.^22^ Smaller series or individual case reports similarly describe either no benefit after quinacrine treatment alone or in combination with chlorpromazine^23^, ^24^, ^25^ or only a transient clinical response with quinacrine.^26^, ^27^, ^28^ A transient response was noted in four of the 40 patients receiving the drug in our study. A more complex analysis of the secondary endpoints is underway. Transient clinical responses have been reported in individual patients treated with amantadine, vidarabine, or levetiracetam, and might be due to symptomatic effects including non-specific arousal, or suppression of spasticity and myoclonus.^29^, ^30^, ^31^ No drug prevents disease progression, although a slowed rate of cognitive deterioration but no effect on survival was recorded in 13 patients treated with flupirtine compared with placebo in the only randomised double-blind trial in human prion disease.^21^

One explanation for the lack of benefit from quinacrine in our study is that adequate drug concentrations were not achieved at the sites crucial for the antiprion effect shown in cell models. A 0·3 μmol/L concentration of quinacrine, estimated to produce half-maximally effective inhibition of PrP^Sc^ accumulation in cell models, corresponds to a concentration of about 120 ng/mL.^7^, ^32^ We did not have serum or CSF concentrations of quinacrine in this trial, but work related to the treatment of malaria suggested steady-state serum concentrations of 30–75 ng/mL in patients taking 300 mg quinacrine per day.^33^ Animal studies showed much lower CSF concentrations but with evidence of accumulation in brain tissue and lysosomes to concentrations matching those seen in infected ScN2a cell culture.^34^, ^35^, ^36^

Our experience highlights several challenges to the assessment of new treatments for human prion disease. First, the rarity of human prion disease emphasises the importance of designing trials that are acceptable to as many patients as possible to maximise the information gained on new drugs. If randomisation that includes a group not receiving the new drug is not acceptable to patients or their carers, other options, such as patient-preference trials or comparisons within patients—both much more difficult to interpret than randomised trials—might be the only options. Incorporation of home visits is crucial for acceptability to patients with prion diseases. Furthermore, the small numbers of patients coupled with the subjectivity of many of the neurological rating scales means that restricting the number of practitioners making assessments is essential to reduce interobserver variability. Second, many patients were identified only when disease was already advanced. Earlier diagnosis is a high priority if patients are to be included in treatment trials, as those with mild to moderate disease are probably most likely to accept randomisation. In this regard, the sensitivity and specificity of clinical investigations including CSF and other markers of early disease need to be more accurately established. New MRI techniques, particularly diffusion-weighted imaging and fluid-attenuated inversion-recovery sequences, offer increased sensitivity in neuroimaging of suspected disease and might usefully be incorporated in diagnostic criteria and criteria defining response to new treatments. Because patients with prion disease form a heterogeneous population and survival is likely to be strongly influenced by other medical interventions, future treatments should be assessed primarily through randomised and adequately controlled clinical trials or at least in prospectively followed cohort studies. Unless a new treatment has a substantial effect on clinical response or survival, comparison with historical or other similar controls will probably not be sufficient to provide proof of efficacy, and under these circumstances open-label uncontrolled studies provide limited information on toxicity at best.
